# The relationship between the number of stenotic coronary arteries and the gut microbiome in coronary heart disease patients

**DOI:** 10.3389/fcimb.2022.903828

**Published:** 2022-08-26

**Authors:** Hao Yu, Le Li, Yu Deng, Guolan Zhang, Mimi Jiang, He Huang, Cheng Li, Zhiyu Lv, Yingshun Zhou, Xing Liu

**Affiliations:** ^1^ Department of Cardiology, The Affiliated Hospital of Southwest Medical University, Luzhou, China; ^2^ Department of Pediatrics, The Affiliated Hospital of Southwest Medical University, Luzhou, China; ^3^ Department of Neurology, The Affiliated Hospital of Southwest Medical University, Luzhou, China; ^4^ Department of Pathogen Biology, The public platform of the Pathogen Biology technology, School of Basic Medicine, Southwest Medical University, Luzhou, China; ^5^ Collaborative Innovation Center for Prevention and Treatment of Cardiovascular Disease of Sichuan Province; Southwest Medical University, Luzhou, China

**Keywords:** coronary heart disease, stenotic coronary arteries, gut microbiome, 16S rRNA sequencing, metabolites, functional prediction

## Abstract

An increasing number of studies have shown that the gut microbiome plays an important role in the development of coronary heart disease (CHD). However, there are no clear studies on the relationship between the gut microbiome and the number of stenotic coronary arteries. To clarify whether the gut microbiome is associated with the number of stenotic coronary arteries in CHD, we performed the 16S rRNA gene sequencing for the V3-V4 region in the gut microbiota from 9 healthy controls (C) and 36 CHD patients, which including 25 CHD patients with multivessel (MV) lesion and 11 CHD patients with single-vessel (SV) lesion. It showed that the abundance of the genus *Escherichia-Shigella* was significantly increased in the MV and SV groups compared with C group, while the abundance of the genera *Subdoligranulum* and *Collinsella* was significantly decreased. Biomarkers based on three gut microbiotas (*Escherichia-Shigella, Subdoligranulum*, and *Collinsella*) and three plasma metabolites(left atrial diameter (LA), low density lipoprotein (LDL), and total bile acids (TBA)) were able to distinguish CHD patients with different numbers of stenotic coronary arteries. Functional prediction of the gut microbiome was performed based on the Kyoto Encyclopedia of Genes and Genomes (KEGG) database. The results showed that the gut microbial function of MV and SV group patients was richer than C group in betaine biosynthesis and unsaturated fatty acid biosynthesis, in the contrast less than C group in sphingolipid metabolism and primary bile acid biosynthesis. In summary, our study showed that the composition and function of the gut microbiome changed significantly from healthy controls to CHD patients with different numbers of coronary lesions.

## Introduction

Coronary heart disease (CHD) is defined as cardiac dysfunction and/or organic disease caused by insufficient myocardial blood supply due to coronary artery stenosis. Atherosclerosis (AS) is the most common cause of coronary artery stenosis. More diseased coronary arteries caused worse outcome in patients. Cohort studies have shown that the number of coronary with ≥75% lumen obstruction is independently associated with an increased risk of 1-year mortality and major adverse cardiovascular events (Ndrepepa et al., 2012). An increasing number of studies have shown significant changes in the composition and function of the gut microbiome in patients with CHD. Koren et al. identified *Chryseomonas, Veillonella*, and *Streptococcus* in AS plaque samples, and some gut microbiome are common to atherosclerotic plaques and are correlated with cholesterol levels (Koren et al., 2011). A metagenome-wide association study showed that the atherosclerotic cardiovascular disease (ACVD) gut microbiome deviates from the healthy status by increased abundance of *Enterobacteriaceae* and *Streptococcus* spp. (Jie et al., 2017). Liu et al. found that the bacterial co-abundance group (CAG), represented by *Roseburia, Klebsiella, Clostridium IV*, and *Ruminococcaceae*, was enriched in the gut microbiota of samples with CHD and had characteristic changes at different stages of CHD (Liu et al., 2019). CHD can be categorized as either acute coronary syndromes (ACS) or chronic coronary syndromes (CCS) according to the severity of clinical symptoms (Knuuti et al., 2020). Previous study showed that that the CCS group experienced a significantly higher ratio of Firmicutes/Bacteroidetes compared with the control group (Sawicka-Smiarowska et al., 2021).A new study shows that ACS patients had distinct serum metabolome and gut microbial signatures as compared with control individuals, and were depleted in a previously unknown bacterial species of the *Clostridiaceae* family (Talmor-Barkan et al., 2022). The relationship between the alteration of microbiota and patients with CHD is not only correlation but also causality (Kwon et al., 2022). Several studies have shown that the occurrence and clinical classification of CHD are related to the changes of gut microbiome, but it remains to be determined whether these changes can affect the number of stenotic coronary arteries.

To address the above questions, we analyzed the gut microbial characteristics of 45 hospitalized patients who had undergone coronary angiography through high-throughput sequencing, including 36 CHD patients (including multivessel (MV) group N=25, single-vessel (SV) group N=11) and 9 healthy controls. Based on 16S rRNA V3-V4 region sequencing and statistical analysis of clinical features, we identified the gut microbiota and clinical features associated with an increased number of stenotic coronary arteries in CHD and further established relationships. This information may contribute to construct a disease classifier to distinguish healthy controls from patients with CHD with different numbers of coronary stenotic lesions.

## Materials and methods

### Ethics statement

The studies involving human participants were reviewed and approved by The Ethics Committee of The Affiliated Hospital of Southwest Medical University. (Approval no. KY2022104). All subjects were voluntarily recruited and informed of the nature of the study before sample collection. Written informed consent was obtained from all study subjects.

### Study design and recruitment

We recruited 45 patients who had undergone coronary angiography at the Affiliated Hospital of Southwest Medical University. We took 9 people with no coronary plaques and smooth intima as the control (C) group; subject with more than 75% stenosis of any of coronary arteries or branches was regarded as the CHD group: 1) 11 people with only one coronary artery stenosis degree ≥75% and other coronary intima completely smooth were regarded as the single-vessel (SV) disease group; 2) 25 people with 2 or more coronary arteries with a stenosis degree ≥75% were regarded as the multivessel (MV) disease group. Patients were excluded if they had any gastrointestinal disease, had a history of gastrointestinal surgery in one year, or had used gut microbiome preparations or antibiotics in the past 1 month. All patients’ feces were collected at the time of their initial appointment ensuring they haven’t eaten lipid-lowering and antiplatelet drugs, which may prevent coronary atherosclerotic. Fresh feces were collected from each subject on the day following coronary angiography, and all collected samples were transported immediately to the laboratory and stored at -80°C.

### DNA extraction and 16S rRNA gene V3-V4 region sequencing

Bacterial DNA was isolated from fecal samples by bead milling for DNA extraction (Godon et al., 1997) and sequenced of the V3-V4 region of the 16S rRNA gene. Deoxyribonucleic acid extracted from each sample was used as a template to amplify the V3-V4 region of the 16S rRNA gene with PCR. Polymerase chain reaction amplification, polymerase chain reaction amplicon sequencing, and quality control of the raw data were performed (Zhang et al., 2010). Sequencing libraries of the V3-V4 region of the 16S rRNA gene were prepared by mixing the purified products in equal proportions for sequencing using the Illumina MiSeq system (Illumina, USA) to generate 100 bp paired-end reads in the forwards and reverse directions (Zhang et al., 2016).

### Sequencing data analysis

Operational taxonomic units (OTUs) were clustered at the cutoff of 97% by using USEARCH v.8.0 (Edgar, 2013). The protocol can be found on the website (http://drive5.com/usearch/manual/uparse_pipeline.html). By comparison with the Silva database (Release138, http://www.arb-silva.de), the RDP classifier (RDP database version 11.5, http://rdp.cme.msu.edu/classifier/classifier.jsp) Bayesian algorithm was used to taxonomically analyze the OTU representative sequences at a 97% similarity level, and the community species composition of each sample was counted at each taxonomic level: domain, kingdom, phylum, class, order, family, genus, and species. The taxonomic composition of each group was visualized as a stacked bar plot at the phylum level and genus level with the ggplot2 package. The QIIME platform (http://qiime.org/scripts/assign_taxonomy.html) was used for alpha and beta diversity analysis. The Shannon index, observed OTUs, and Simpson index were evaluated. Beta diversity analysis was performed using standardized OTU abundance tables, including principal component analysis (PCA) and principal coordinate analysis (PCoA) based on Bray-Curtis distance. Partial least-squares discrimination analysis (PLS-DA) and analysis of similarities (ANOSIM) were used to test for statistical significance among the three groups. Wherever mentioned, the Benjamini-Hochberg method was used to control the false discovery rate (FDR). Data visualization was achieved using the vegan package and the mixOmics package of the R language. One-way analysis of variance (ANOVA) and Kruskal-Wallis test were used to find differential species between groups. Linear discriminant analysis Effect Size (LEfSe, http://huttenhower.sph.harvard.edu/galaxy/root?tool_id=lefse_upload) was used to find communities or species that had significant differential effects on grouping. Data visualization was achieved using the stats package for R. DESeq2 was utilized to identify significantly differential features, and the Benjamini-Hochberg method was used to control the FDR. Phylogenetic Investigation of Communities by Reconstruction of Unobserved States (PICRUSt2) was utilized to predict the functional compositions. Pathways that were different in abundance among the C, SV and MV groups were obtained using Welch’s t-test by STAMP software (v2.1.3). The visualization of the identified pathways was obtained by using the pheatmap package. To obtain functional predictions based on the 16S rRNA sequences, the taxonomic classification of sequences was performed based on the Kyoto Encyclopedia of Genes and Genomes (KEGG,http://www.genome.jp/kegg/).

### Spearman multi-omic correlation analysis

Spearman correlations among important bacterial taxa, clinical features and metabolic pathway were calculated by using SPSS 23.0. The correlations between features were visualized using the pheatmap package.

### Enterotype analysis

According to the relative abundance of bacteria at the genus level, the Bray-Curtis distance was calculated, and PAM (Partitioning Around Medoids) clustering was performed. Then, the optimal cluster K value was calculated by the Calinski-Harabasz (CH) index. Finally, principal coordinates analysis (PCoA, K ≥ 2) was used for visualization. Data analysis and visualization were performed using the R package ade4 package, cluster package, and clusterSim. At the same time, based on the species abundance information and the results of enterotype analysis, species with significant differences among enterotypes were identified by statistical testing. Species with significant differences and the highest relative abundance among enterotypes were defined as the name of the enterotype.

## Results

### Clinical characteristics of the study participants

To identify the relationship between the number of stenotic coronary arteries and gut microbiota, we collected feces from 45 patients who underwent coronary angiography at the Affiliated Hospital of Southwest Medical University (detailed in the “Materials and methods” section) and divided them into a control group (C) (n = 9), a MV group (n = 25), and a SV group (n = 11). The characteristics and traditional cardiovascular risk factors for the participants were summarized in **Table 1**. Left ventricular ejection fraction (LVEF) and left ventricular end diastolic diameter (LVDd) are the most important indicators to evaluate left ventricular function. Right ventricular diameter (RV) can be used to assess right ventricular function. N-terminal pro-B type natriuretic peptide (NT-proBNP) is an index for the overall evaluation of cardiac function. We also counted NT-proBNP, LVEF, LVDd, RV and other indicators in **Table 1** to analyze the differences in cardiac function of patients in each group as a whole. Except for the significant differences in the triglyceride (TG), high density lipoprotein (HDL) and LVDd levels between C vs. SV and C vs. MV (P < 0.05), other clinical features showed no significant difference between the C and CHD subgroups. However, the LVDd of patients in MV group and SV group is basically within the normal range. Therefore, we supposed that the number of coronary stenotic vessels in patients with CHD is only affected by the differences in the composition and structure of the gut microbiota.

**Table 1:** Clinical characteristics of study participants.

	C (n=9)	MV (n=25)	SV (n=11)	P value
age (years)^#^	61.56 ± 8.59	61.2 ± 10.83	58.73 ± 11.82	0.79
Female^&^	4 (44.4)	9 (36)	2 (18.2)	0.42
Smoking history&	3 (33.3)	12 (48)	9 (81.2)	0.07
Hypertension history&	4 (44.4)	13 (52)	6 (54.5)	0.90
Diabetes history&	3 (33.3)	12 (48)	3 (27.3)	0.46
NT-proBNP(pg/ml)^#^	759.67 ± 1268.76	1490.30 ± 2128.41	301.27 ± 649.84	0.15
LVEF(%)^#^	56.56 ± 10.64	61.68 ± 6.01	61.91 ± 6.92	0.18
LVDd(mm)^#^	51.33 ± 6.67	47.04 ± 3.98	44.09 ± 5.66	0.01^§^
LVDs(mm)^#^	34.11 ± 7.75	27.79 ± 11.01	28.64 ± 4.88	0.22
LA(mm)^#^	33.56 ± 6.46	32.12 ± 4.87	29.18 ± 3.03	0.12
IVS(mm)^#^	10.00 ± 1.50	12.12 ± 5.89	10.73 ± 1.01	0.43
RV(mm)^#^	21.22 ± 2.22	20.96 ± 1.74	20.82 ± 1.47	0.88
TC(mmol/L)^#^	4.85 ± 1.14	5.36 ± 1.42	4.43 ± 0.94	0.13
TG(mmol/L)^α^	0.65 (0.83,1.23)	1.26 (1.79,2.46)	1.19 (1.92,3.72)	0.03^*^§^^
HDL(mmol/L)^#^	1.34 ± 0.26	1.07 ± 0.2	1.09 ± 0.17	0.01^*^§^^
LDL(mmol/L)^α^	1.11 (1.28,1.58)	0.98 (1.06,1.19)	0.94 (1.13,1.19)	0.13
BMI(kg/m^2^)^#^	22.84 ± 3.06	23.29 ± 3.13	24.07 ± 3.5	0.68
ALT(U/L)^α^	13.45 (19.2,35.9)	18.45 (24.7,46.6)	15.9 (27.4,40.3)	0.34
AST(U/L)^α^	18.55 (23.4,31.55)	20.15 (30.5,87.45)	16.8 (21.7,26.7)	0.08
TP(g/L)^#^	72.08 ± 7.35	69.26 ± 6.23	68.48 ± 6.7	0.44
ALB(g/L)^#^	44.52 ± 2.95	42.63 ± 4.15	42.66 ± 7.17	0.59
TBil(umol/L)^#^	13.4 ± 4.2	13.26 ± 6.44	11.94 ± 2.69	0.76
TBA(umol/L)^α^	3.15 (5.3,8.8)	2 (3.6,6.4)	2.8 (3.9,5.1)	0.35
UA(umol/L)^#^	364.43 ± 108.5	356.51 ± 98.34	370.92 ± 69.6	0.91
Crea(umol/L)^#^	65.83 ± 16.12	68.74 ± 16.94	68.21 ± 11.67	0.89
GFR(ml/min)^α^	79.35 (102.5,107.1)	91.25 (98.2,107.35)	89.1 (103.3,107.6)	0.96
WBC (10^9/L)^α^	4.99 (7.16,7.48)	7.14 (8.33,10.82)	5.73 (7.06,7.96)	0.05
NEU (10^9/L)^α^	2.5 (4.16,6.04)	4.34 (5.74,9.27)	4.11 (4.43,5.34)	0.11
LYM (10^9/L)^#^	1.62 ± 0.79	1.72 ± 0.7	1.54 ± 0.38	0.73
MONO (10^9/L)^α^	0.27 (0.35,0.46)	0.29 (0.38,0.57)	0.29 (0.33,0.44)	0.86
EOS (10^9/L)^α^	0.04 (0.15,0.29)	0.04 (0.12,0.22)	0.02 (0.06,0.1)	0.26
BASO (10^9/L)^α^	0.01 (0.03,0.04)	0.02 (0.03,0.05)	0.01 (0.02,0.03)	0.17
NEU-R(%)^#^	64.36 ± 14.24	70.26 ± 12.59	71.32 ± 8.56	0.38
RBC (10^12/L)^#^	4.52 ± 0.44	4.44 ± 0.45	4.75 ± 0.67	0.26
HB (g/L)^#^	133.89 ± 12.5	135.32 ± 14.56	144.45 ± 15.21	0.17
HCT^#^	0.41 ± 0.03	0.41 ± 0.04	0.43 ± 0.04	0.28
PLT (10^9/L)^#^	232 ± 62.18	208.76 ± 55.28	208.55 ± 71.27	0.59
HbA1c (%)^α^	4.78 (5.09,7.55)	5.43 (5.9,7.15)	5.34(5.64,6.2)	0.22

^#^mean ± SD,^&^n (%),^α^median (IQR); Continuous, normally distributed variables among the three groups were analyzed by a one-way analysis of variance. The Kruskal-Wallis H-test was applied for data of this type that were not normally distributed. Continuous, normally distributed variables between two groups were analyzed by Student’s t-test. The Mann Whitney U test was applied for data of this type that were not normally distributed. Categorical variables were compared by the χ^2^-test. NT-proBNP, N-terminal pro-B type natriuretic peptide; LVEF, left ventricular ejection fraction; LVDd, left ventricular end diastolic diameter; LVDs, left ventricular end diastolic diameter; LA, left atrial diameter; IVS, Interventricular septum; RV, right ventricular diameter; TC, total cholesterol; TG, triglyceride; HDL, high-density lipoprotein; LDL, low density lipoprotein; BMI, body mass index; ALT, alanine aminotransferase; AST, aspartate aminotransferase; TP, total protein; ALB, albumin; TBIL, total bile acids; TBA, total bile acids; UA, uric acid; Crea, creatinine; GFR, glomerular filtration rate; WBC, white blood cell; NEU, neutrophil; LYM, lymphocyte; MONO, mononuclear; EOS, eosinophils; BASO basophils; NEU-R, neutrophil rate; RBC, red blood cell; HB, hemoglobin; HCT, hematocrit; PLT, platelets; HbA1c, glycated hemoglobin. *P<0.05 for equality between C vs MV;^§^P<0.05 for equality between C vs SV.

### Overview of the 16S rRNA gene sequencing data

**Figure 1:**
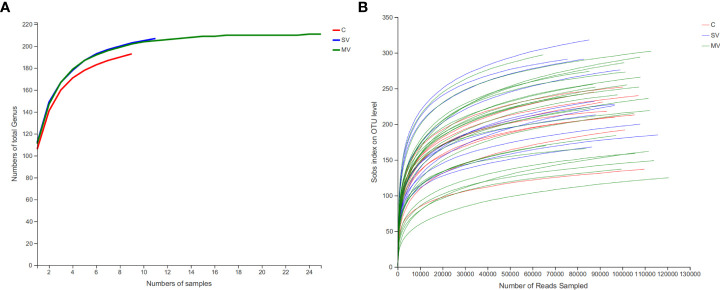
Coverage of members in the gut microbiota of each group subject. **(A)** Rarefaction curves of detected gut microbiota genus of patient in each group reach the saturation stage with increasing numbers of samples, indicating that the gut microbiota in our population capture most gut microbiota members of human. **(B)** Rarefaction curves of detected bacterial OTUs of the gut microbiota from each group subject reach saturation stage with increasing sequencing depth.

We sequenced the V3-V4 region of the gut microbiome 16S rRNA gene in 9 healthy controls (C), 25 patients with multivessel coronary artery disease (MV) CHD, and 11 patients with single-vessel coronary artery disease (SV) CHD (sequences were detailed in Supplementary Data 1). After quality control and removal of human DNA, a total of 5,396,535 sequences were obtained, with an average of 119,923 sequences per sample and an average length of 417 bp per sample sequence. Using the Silva database (Release138http://www.arb-silva.de), reads from all samples were species annotated, and species clustering was performed according to 97% similarity, resulting in a total of 1002 OTUs. Then, we clustered the OTUs at the genus level, resulting in 306 gut microbiota genera. After excluding the genera with an average abundance contributing with less than 0.01% to the total, 211 genera were used for the subsequent analyses (**Supplementary Table 1**). Rarefaction analysis shows that observed-genera numbers tend to stabilize when the sample size of each group reaches 10 (**Figure 1A**). The revealed that the gut microbiota in our population capture most gut microbiota members of human. At the same time, rarefaction analysis also shows that the number of OTUs observed also tends to be stable when the sequence reads reaches 60000, indicating the sequencing depth is sufficient (**Figure 1B**). At the phylum level, the gut microbiota of all subjects was mainly classified into five phyla: *Firmicutes, Bacteroides, Proteobacteria, Actinobacteriota*, and *Verrucomicrobiota*. At the genus level, the gut microbiota was mainly composed of *Bacteroides, Blautia, Escherichia-Shigella, Faecalibacterium*, and *Streptococcus* (**Supplementary Figure S1**).

### Microbial strains associated with CHD

**Figure 2:**
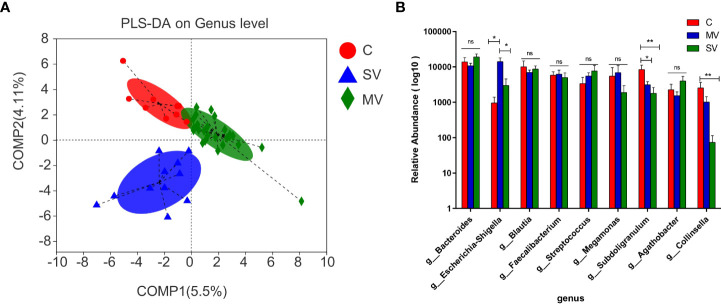
Difference test and significant different genera among groups microbiome. **(A)** Partial least-square discrimination analysis (PLS-DA). **(B)** The Kruskal-Wallis test was applied for the difference of gut microbiota among three groups. Tukey test was performed followup tests between any two groups. *P values < 0.05, **P values < 0.01, ns, no significance.

Alpha diversity analysis showed no significant difference among three groups in either gene richness or diversity (**Supplementary Figure S2**). As seen by principal component analysis (PCA) and principal coordinates analysis (PCoA), the distribution of gut microbiota among the three groups showed a tendency to separate (**Supplementary Figure S3**), indicating that the flora among the three groups was evidently different. We further showed differences in microbiota among the three groups by PLS-DA (**Figure 2A**). The Kruskal-Wallis test was performed to identify differential species of each group. The results showed that the *Subdoligranulum* and *Collinsella* genera were significantly more abundant in group C subjects than in the SV and MV groups (MV vs. C, P < 0.05; MV vs. SV, P < 0.01). At the same time, the abundance of the *Escherichia-Shigella* genus was significantly higher in subjects in the MV group than in those in the C and SV groups (**Figure 2B**) (C vs. MV, P < 0.05; C vs. SV, P < 0.01).

**Figure 3:**
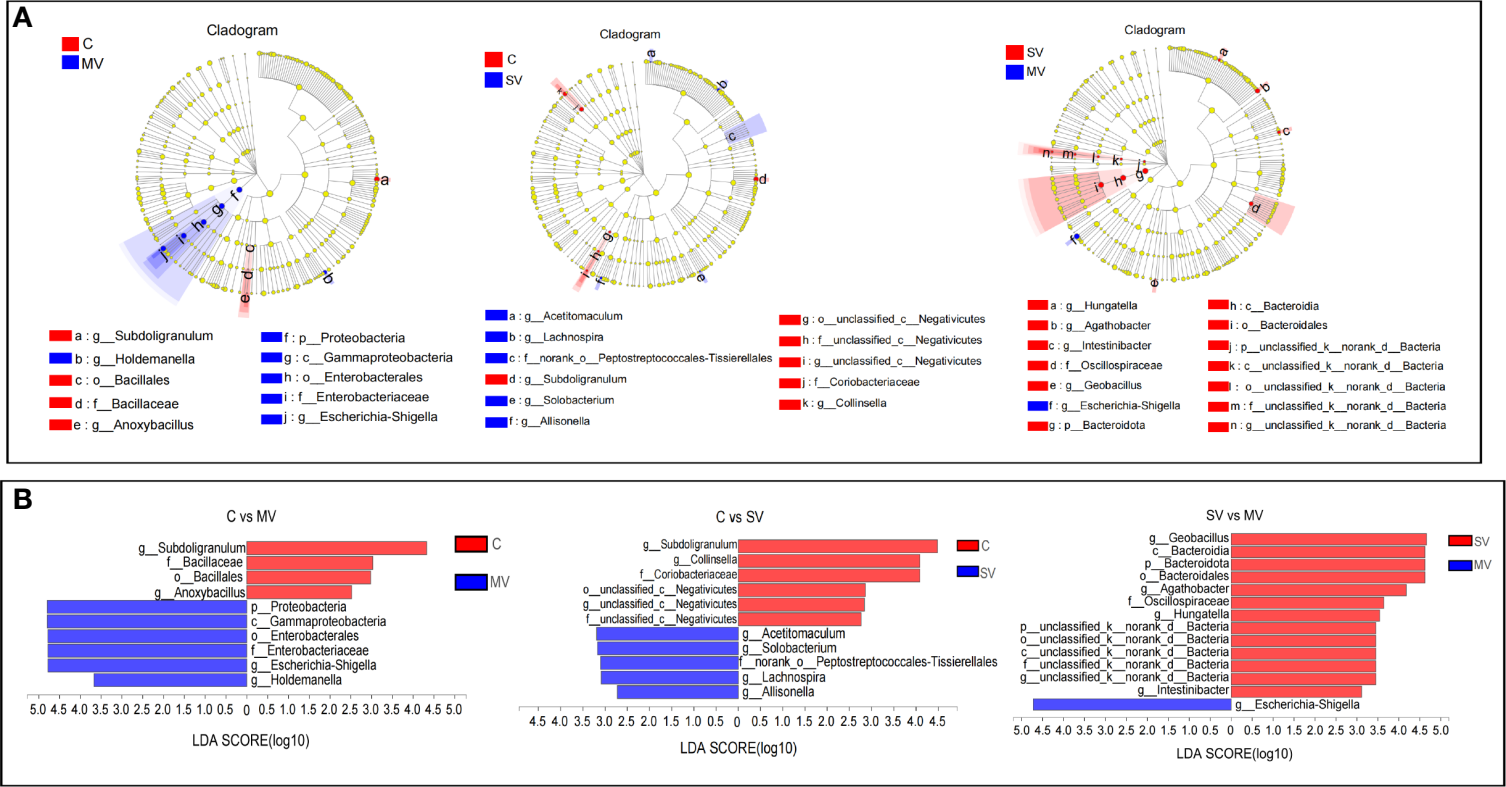
Linear discriminant analysis effect size (LEfSe) analysis of species differences. The non-parametricfactor Kruskal-Wallis **(K-W)** sum-rank test was used to detect characteristics of significant abundance differences and to find classes that were significantly different from abundance. Linear discriminant analysis (LDA) was employed to estimate the magnitude of the effect of each component (species) abundance on the differential effect. **(A)** Hierarchical dendrogram of multilevel species. **(B)** Linear discriminant analysis (LDA).

Next, we used linear discriminant analysis (LDA) to identify the gut microbiota that affected grouping differences (**Figure 3**). The results showed that *Escherichia-Shigella* and *Subdoligranulum* contributed the most to the difference in C vs. MV, *Collinsella*, and *Subdoligranulum* contributed the most to the difference in C vs. SV, and *Geobacillu*s and *Escherichia-Shigella* contributed the most to the difference in SV vs. MV. At the bacterial phylum level, *Proteobacteria* was the main phylum distinguishing the C vs. MV group, *Bacteroides* was the main phylum distinguishing the SV vs. MV group, while the C vs. SV group had no significant difference at the phylum level. Our study showed that enrichment of *Escherichia-Shigella* was associated with multivessel coronary stenosis. In contrast, *Subdoligranulum* and *Collinsella* may be associated with normal coronary arteries.

### Enterotypes in the cohort

It has been shown that normal human gut microbiota can be divided into three types: *Bacteroides* enrichment leads to enterotype 1, *Prevotella* enrichment leads to enterotype 2, and *Ruminococcus* enrichment leads to enterotype 3 (Arumugam et al., 2011). We calculated the Bray-Curtis distance of genus abundances to cluster the samples, and the Calinski-Harabasz index indicated that the optimal number of clusters was five (**Supplementary Figure S4**). The five enterotypes we observed had different contributors at the genus level: *Bacteroides* resulted in enterotype 1 and enterotype 2, *Megamonas* resulted in enterotype 3, *Lactobacillus* resulted in enterotype 4, and *Escherichia-Shigella* resulted in enterotype 5 (**Supplementary Figure S4**). At the same time, we found that enterotype 5 was composed of only SV and MV group samples, so we speculated that enterotype 5, characterized by enrichment of *Escherichia-Shigella*, may be associated with the occurrence of CHD.

### Links between the gut microbiome and clinical features of CHD

**Figure 4:**
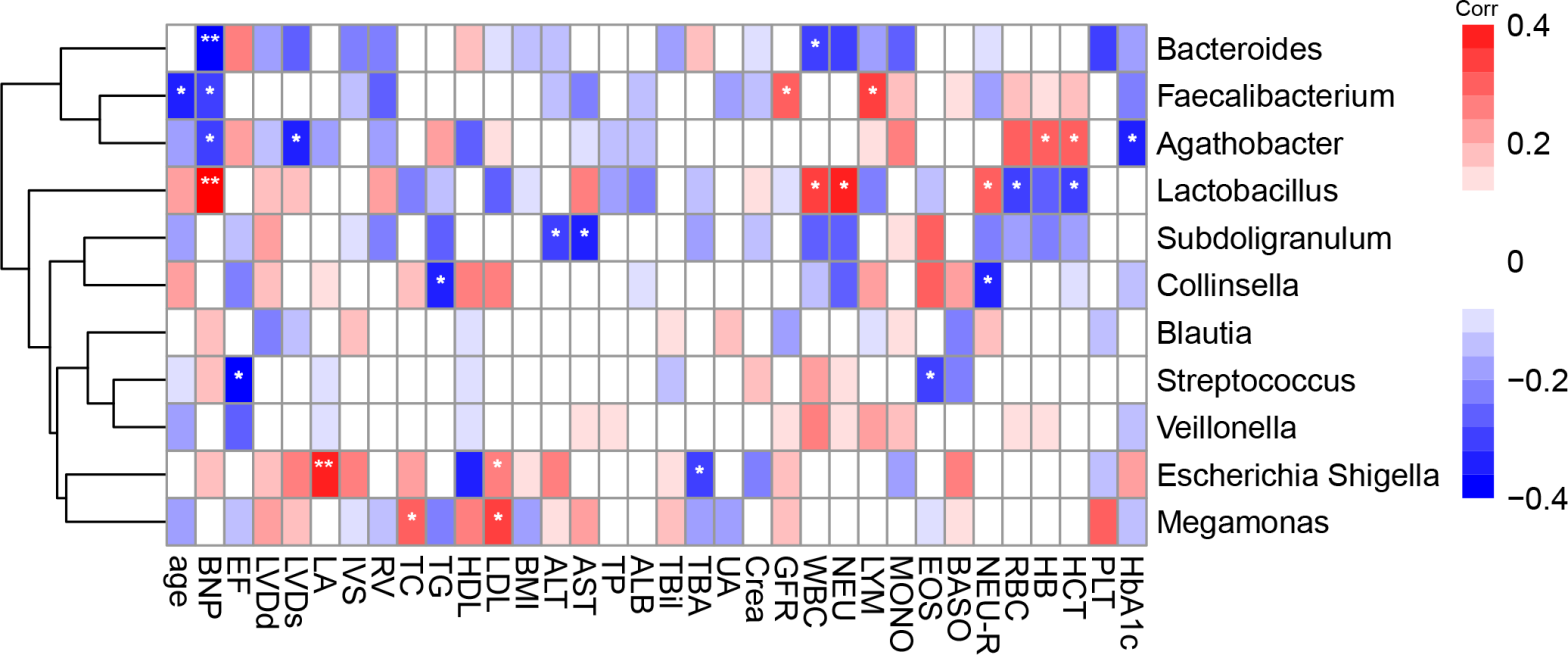
Spearman correlations between gut microbiota genus and clinical indicators. The colour represents positive (red) or negative (blue) correlations, and FDRs are denoted as follows: *FDR < 0.05, **FDR < 0.01. NT –proBNP, N-terminal pro-B type natriuretic peptide; LVEF, left ventricular ejection fraction; LVDd; left ventricular end diastolic diameter; LVDS, left ventricular end diastolic diameter; LA, left atrial diameter; IVS, Interventricular septum; RV, right ventricular diameter; TC, total cholesterol; TG, triglyceride; HDL, high-density lipoprotein; LDL, low density lipoprotein; BMI, body mass index; ALT, alanine aminotransferase; AST, aspartate aminotransferase; TP, total protein; ALB, albumin; TBIL, total bile acids; TBA, total bile acids; UA, uric acid; Crea, creatinine; GFR, glomerular filtration rate; WBC, white blood cell; NEU, neutrophil; LYM, lymphocyte; MONO, mononuclear; EOS, eosinophils; BASO, basophils; NEU-R, neutrophil rate; RBC, red blood cell; HB, haemoglobin; HCT, haematocrit; PLT, platelets; HbA1c, glycated haemoglobin.

We calculated the Spearman correlation coefficient between a range of clinical indicators that may be associated with the onset of CHD (**Table 1**) and gut microbiota in the top 10 most abundant genera and *Collinsella* genus (**Figure 4**). We found that the genus *Escherichia-Shigella* was positively correlated with plasma low density lipoprotein (LDL) and left atrial diameter (LA), and negatively correlated with total bile acids (TBA); The genus *Collinsella* was negatively correlated with neutrophil ratio (NEU-R) and TG, and the genus *Subdoligranulum* was negatively correlated with alanine aminotransferase (ALT) and aspartate aminotransferase (AST). In addition, the genus *Megamonas* was positively correlated with LDL and total cholesterol (TC).

### Combination of gut microbiota and clinical features provides an effective biomarker set to distinguish three group subjects

**Figure 5:**
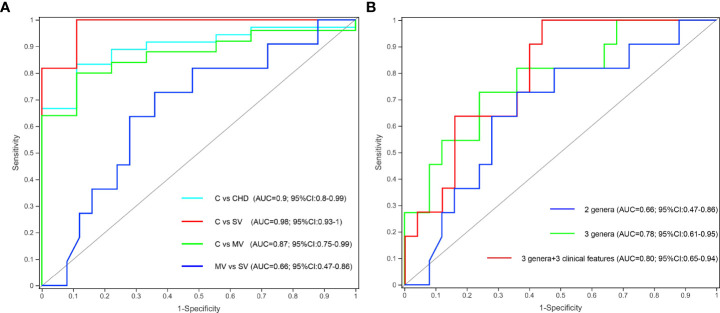
Gut microbiota and clinical features could effectively distinguish C from CHD, SV, and MV group subjects. **(A)** Two specific genera to build the prediction model yielded an AUC based on ROC analysis. **(B)** Gut microbiota clinical features to build the prediction model yielded an AUC based on ROC analysis.

To determine whether gut microbiota and clinical indicators can be regarded as biomarkers to distinguish the number of stenotic coronary arteries in patients with CHD, we constructed a few prediction models based on gut microbiota and clinical features as mentioned above. The Kruskal-Wallis test showed that the *Subdoligranulum* and *Collinsella* genera were significantly more abundant in group C subjects than in the SV and MV groups, and the abundance of the *Escherichia-Shigella* genus was significantly higher in the MV group subjects than in the C and SV groups. We finally selected *Subdoligranulum* and *Collinsella* genera as a gut bacterial biomarker set for controls. Receiver operating characteristic (ROC) analysis revealed that this gut bacterial set could distinguish C from CHD, SV, and MV with area under the curve (AUC) values of 0.9, 0.98, and 0.87, respectively (**Figure 5A**). However, the predictive potential of this biomarker set for SV and MV groups is low. Spearman correlation coefficient analysis showed that the genus *Escherichia-Shigella* was positively correlated with LDL and LA, and negatively correlated with TBA. Therefore, we added *Escherichia-Shigella* and three clinical features (LDL, LA, and TBA) to construct a new prediction model to distinguish SV and MV groups. The result revealed the new biomarker set exhibited a higher predicting potential to distinguish SV from MV patients (AUC 0.66 vs 0.80) (**Figure 5B**).

### Changes in gut microbiome function in CHD patients

**Figure 6:**
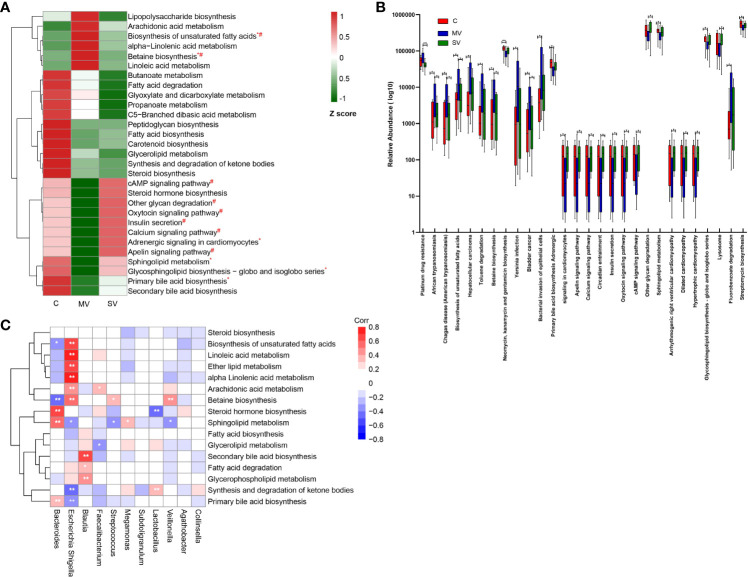
Changes in gut microbiome function in CHD patients. **(A)** Relative abundance of part of bacterial metabolic pathways in different groups. The abundance profiles were transformed into Z scores by substracting the average abundances and dividing the standard deviations of all the samples. The Z score was negative (shown in green) when the row abundance was lower than the mean. Statistical analysis of metabolic pathways was performed using the Kruskal-Wallis test. *P<0.05 for equality between C vs MV: #P<0.05 for equality between MV vs SV. **(B)** The box plot shows that metabolism pathway (KEGG pathway level 3) significantly changed between different groups by Kruskal-Wallis test. *, Kruskal-Wallis test P-values<0.05; **, Kruskal-Wallis test P-values<0.01, boxes represent the inter-quartile ranges, and lines inside the boxes denote medians. **(C)** Spearman correlations between gut microbiota genera and functions. The colour represents positive (red) or negative (blue) correlations, and FDRs are denoted as follows: *FDR < 0.05, **FDR < 0.01.

To identify the gut microbiome functional changes in CHD patients with different numbers of coronary lesions, we first functionally annotated all sample sequences using the KEGG database (**Supplementary Table 2**). We focused on the metabolic pathways of gut microbiota and focused on lipid, carbohydrate, and glycan metabolism. We normalized the resulting functional annotation table for abundance. The results showed that the enrichment of metabolic pathways such as biosynthesis of unsaturated fatty acids, α-linolenic acid metabolism, betaine biosynthesis, and linoleic acid metabolism showed a trend of MV > SV > C group. Enrichment of metabolic pathways such as sphingolipid metabolism, glycosphingolipid biosynthesis, primary bile acid biosynthesis, and secondary bile acid biosynthesis showed the C > SV > MV group (**Figure 6A**). In addition, there were also many metabolic pathways with reduced enrichment in the CHD group, such as butyrate metabolism, fatty acid degradation, glyoxylate, and dicarboxylic acid metabolism, propionate metabolism, C5 - branched-chain dibasic acid metabolism, peptidoglycan biosynthesis, fatty acid biosynthesis, carotenoid biosynthesis, glyceride metabolism, synthesis and degradation of ketones, and steroid biosynthesis etc.

In order to further clarify which gut microbiota function may be related to the occurrence of CHD and lead to different numbers of coronary artery stenoses, we performed Kruskal-Wallis tests of the functional annotation table. We found that the group C gut microbiota was significantly superior to the SV and MV groups in sphingolipid metabolism and primary bile acid biosynthesis (**Figure 6B**) (P < 0.05). In contrast, the gut microbiota was clearly dominant in MV group patients in terms of betaine biosynthesis and biosynthesis of unsaturated fatty acids (**Figure 6B**) (P < 0.05). In addition, spearman correlation between gut microbiota and function indicated that *Escherichia-Shigella* was negatively correlated with sphingolipid metabolism and primary bile acid biosynthesis, and positively correlated with betaine biosynthesis and biosynthesis of unsaturated fatty acids (**Figure 6C**). The result indicates that *Escherichia-Shigella* may affect the metabolism of host to lead to the occurrence and development of coronary heart disease. In conclusion, our study showed that gut microbial function was markedly altered in patients with CHD compared to controls. At the same time, the functional changes in the gut microbiota were more obvious in patients in MV group.

## Discussion

In the current study, we prove that CHD patients had significant differences in the composition and function of the gut microbiota compared with healthy group and may further change with the number of stenotic coronary arteries. Through 16S rDNA sequencing and determination of clinical indicators, we found that gut microbiota and clinical indicators that exhibited significant changes with an increasing number of stenotic coronary arteries were significantly correlated and might be used independently as biomarkers to distinguish the number of stenotic coronary arteries in CHD patients. In addition, we also found that *Escherichia-Shigella* may affect the biosynthesis of betaine and thus lead to the occurrence and development of coronary heart disease.

Our data indicated that the abundance of *Escherichia-Shigella* was significantly increased in the gut microbiota of patients in the MV group compared with groups C and SV. This indicates that the high abundance of *Escherichia-Shigella* is associated with multivessel coronary artery lesions. Interestingly, enterotype analysis also showed that *Escherichia-Shigella* enterotype was enrichment in CHD patients. *Shigella* genus is defined clinically, and *Escherichia* is defined phylogenetically. *Escherichia-Shigella* is actually a collective name for pathogenic species in the *Shigella* genus. Studies had shown that *Escherichia-Shigella* can cause endotoxemia and systemic inflammation (Lang et al., 2020), and the inflammatory response has been proven to be an important intermediate link in the onset of cardiovascular disease (Durpes et al., 2015; Gao et al., 2016; Herrero-Fernandez et al., 2019; Marchio et al., 2019). These showed that *Escherichia-Shigella* may cause CHD through inflammation, and as the abundance of *Escherichia-Shigella* increases, the number of coronary stenosis lesions also increases. In addition, our research also found that compared with the control group, the abundance of *Collinsella* and *Subdoligranulum* in the gut microbiota of subjects in the MV and SV groups was significantly reduced. This showed that the high abundance of the genera *Collinsella* and *Subdoligranulum* may be associated with milder coronary atherosclerotic lesions. Related studies have shown that prebiotic oligofructose can increase the content of *Collinsella* in the gut microbiota of rats, thereby reducing the content of triglycerides in the plasma to inhibit weight gain in rats (Klancic et al., 2020). A series of studies had shown that an increase in the abundance of *Subdoligranulum* can significantly reduce subjects’ fasting blood glucose, plasma triglyceride content and body fat rate, and reduce the incidence of hyperlipidemia, hyperglycemia, and obesity (Everard et al., 2011; Bajaj et al., 2012; Leclercq et al., 2014; Dao et al., 2016; Louis et al., 2016). However, an animal experiment showed that treating diabetic mice with *Subdoligranulum* can increase the abundance of the gut microbiota *Subdoligranulum*, but there was no significant improvement in the characteristics of diabetes,for instance weight gain, increased fat mass, glucose tolerance, blood lipids, etc. (Van Hul et al., 2020). In other words, although studies had shown that increasing *Subdoligranulum* can improve metabolic diseases such as obesity, hyperlipidemia, and hyperglycemia, the exact physiological role of *Subdoligranulum* in animals or humans is still unclear. By observing that *Collinsella* and *Subdoligranulum* were simultaneously enriched in the C group samples, we boldly hypothesized that *Subdoligranulum* can have beneficial effects on the body when it coexists in large numbers with *Collinsella*. Further animal experiments are required to verify our hypothesizes. In conclusion, our research showed that the composition of the gut microbiota and clinical indicators change with the increase in the number of stenotic coronary arteries in patients with CHD.

One-way ANOVA of clinical characteristics showed that except for the significant differences in the TG, HDL, and LVDd levels between C vs. SV and C vs. MV, other clinical features showed no significant difference between the C and CHD subgroups. It indicated that the number of coronary stenotic vessels in patients with CHD is only affected by the differences in the composition and structure of the gut microbiota. In addition, spearman correlation coefficient analysis showed that the genus *Escherichia-Shigella* was positively correlated with LDL and LA, and negatively correlated with TBA. A new study showed that bile acid could promote intestinal cholesterol absorption and suppress hepatic cholesterol synthesis to prevent hypercholesterolemia (Semova et al., 2022). High levels of LDL are recognized as one of the risk factors leading to the occurrence and development of coronary heart disease. These results suggest that the cause of coronary heart disease caused by *Escherichia-Shigella* may be closely related to host metabolism. We selected *Subdoligranulum* and *Collinsella* genera as a gut bacterial biomarker set to distinguish C from CHD, SV, and MV with AUC values of 0.9, 0.98, and 0.87, respectively, which display a promising prediction utility. Meanwhile, the prediction model constructed by three genera (Subdoligranulum, Collinsella, and Escherichia-Shigella) and three clinical features (LDL, LA, and TBA) distinguished effectively SV and MV groups with AUC 0.80.

The human gut microbiota interacts extensively with the host through metabolic exchange and substrate co-metabolism. Human metabolites are composed of endogenous metabolites, exogenous metabolites, metabolites from the gut microbiota, and cometabolites between bacteria and the host. Changes in the metabolic function of the gut microbiota can predict changes in the metabolic function and metabolites of the host to a certain extent (Kaye et al., 2020). We observed that the gut microbiota of group C was significantly higher than that of the SV group and the MV group in terms of sphingolipid metabolism and primary bile acid biosynthesis, trending to the C>SV>MV group. Sphingolipids are the basic components of cell membranes and organelles and include ceramide, sphingosine 1-phosphate (S1P), sphingosine, sphingomyelin, glycosphingolipids, and other molecules (Maceyka and Spiegel, 2014). Research showed that genes functioning in sphingosine metabolism and the S1P pathway showed drastically increased expression in the germ-free mice (Formes et al., 2021). Ceramide, the core product of sphingolipid metabolism, has long been considered a substance that causes atherosclerosis (Maceyka and Spiegel, 2014). In mouse, rabbit, and rat cardiac ischemia/reperfusion injury (IRI) models, it was found that the infarct site and blood ceramide increased, while S1P decreased (Cui et al., 2004; Knapp et al., 2013; Pan et al., 2014). Studies had shown that ceramide produces oxidative stress in human endothelial cells, thereby reducing biologically active NO. The decrease in the bioavailability of NO aggravates the proatherogenic effect of ceramide (Li et al., 2002). At the same time, many studies have shown that S1P has a significant cardioprotective effect (Keul et al., 2016; Alessenko et al., 2018; Kurano and Yatomi, 2018). The sphingosine 1-phosphate receptor-1 (S1PR1)-specific agonist can alleviate the hypoxic damage of mouse cardiomyocytes. Animal experiments have shown that S1P can induce significant recovery of heart function and reduction of infarction in patients with IRI (Karliner et al., 2001; Vessey et al., 2006; Jin et al., 2007). In addition, an animal experiment showed that pretreatment of animals with sphingosine before ischemia or perfusion can cause a significant reduction in infarct size. This indicates that sphingosine is also a cardioprotective agent in cardiovascular disease (Vessey et al., 2009). However, it is not clear which sphingolipid molecules increase in the host plasma caused by the gut microbiota. Through bile salt hydrolysis and bile acid 7a-dehydroxylation, the gut microbiota affects the host’s physiology and produces a pool of unbound hydrophobic bile acids (secondary bile acids) (Everard et al., 2011). Secondary bile acids (such as deoxycholic acid) can be used as direct antibacterial agents to reduce bacterial translocation and the systemic inflammatory response (Begley et al., 2005; Ubeda et al., 2016). Relevant studies have shown that damage to the vascular endothelial barrier and inflammation are important factors in the formation of coronary atherosclerosis (Durpes et al., 2015; Gao et al., 2016; Herrero-Fernandez et al., 2019; Marchio et al., 2019). Meanwhile, spearman correlation coefficient analysis showed that the genus *Escherichia-Shigella* was negatively correlated with sphingolipid metabolism and primary bile acid biosynthesis. These results indicates that the genus *Escherichia-Shigella* may inhibite sphingolipid metabolism and primary bile acid biosynthesis to exacerbate vascular inflammation and vascular endothelial dysfunction, thus leading to coronary endothelial injury and atherosclerosis. Our research provided basic support for further exploring the influence of gut microbiota on human sphingolipid and bile acid metabolism and its mechanism.

In contrast, the betaine biosynthesis pathway was significantly higher in the MV group than in the C group and SV group. Spearman correlation coefficient analysis showed that the genus *Escherichia-Shigella* was positively correlated with betaine biosynthesis. Betaine is a raw material for the synthesis of trimethylamine oxide (TMAO), which can be converted to TMAO under the action of gut microbiota (Wang et al., 2014). Specifically, endogenous or exogenous betaine produces trimethylamine (TMA) in response to gut microbes, and then host liver flavin monooxygenase (FMO) catalyzes the conversion of TMA to TMAO (Koeth et al., 2013). A large number of studies had shown that TMAO can promote the occurrence and development of atherosclerosis in animal models (Wang et al., 2011; Koeth et al., 2013; Tang et al., 2013). Multiple cohort studies had shown that blood TMAO levels are associated with the risk of coronary atherosclerotic heart disease and major adverse cardiac events (Lever et al., 2014; Mente et al., 2015; Heianza et al., 2020). In addition, an animal study has shown that nonlethal inhibition of the production of trimethylamine by intestinal microbes can treat atherosclerosis (Wang et al., 2015). Taken together, we speculated that *Escherichia-Shigella* may promote plasma TMAO content by promoting betaine biosynthesis, which leads to coronary atherosclerosis.

## Conclusion

In conclusion, this study suggests that the composition and diversity of the gut microbiota change significantly from healthy controls to CHD subgroups with different numbers of coronary lesions. At the same time, we also found several gut microbiotas associated with leading to CHD and affecting the number of coronary lesions. We constructed a disease classifier based on these related gut microbiota and plasma metabolites to distinguish the control group from CHD, providing a new direction for the diagnosis and prognosis of CHD. In addition, our results predict several functional pathways based on gut microbiome information in patients with CHD, which may enhance our understanding of the pathogenesis of CHD. In summary, the changes in gut microbiota structure and function are closely related to the occurrence and development of CHD, and changing the structure and function of gut microbiota may become a new measure to prevent and treat CHD.

## Data availability statement

The datasets presented in this study can be found in online repositories. The names of the repository/repositories and accession number(s) can be found below: https://www.ncbi.nlm.nih.gov/, PRJNA810651.

## Ethics statement

The studies involving human participants were reviewed and approved by The Ethics Committee of The Affiliated Hospital of Southwest Medical University. The patients/participants provided their written informed consent to participate in this study. Written informed consent was obtained from the individual(s) for the publication of any potentially identifiable images or data included in this article.

## Author contributions

HY, YZ and XL: designed the experiments. YD and HH: collected samples. HY, YZ, and LL: analyzed the data. HY, GZ, and MJ: visualization. HY: writing - original draft. LL, XL, ZL, CL, and YZ: writing-review and editing. XL and YZ: project administration. All the authors read and approved the final manuscript. All authors contributed to the article and approved the submitted version.

## Acknowledgments

We thank Dr Mingzhi Liu(Karolinska Institutet) for proofreading. This study was supported by the Collaborative Innovation Center for Prevention and Treatment of Cardiovascular Disease of Sichuan Province (xtcx2016-17). Sichuan Province Science and Technology project (2020YJ0338), Southwest Medical University Foundation (21YYJC0529). Doctoral Research Initiation Fund of Affiliated Hospital of Southwest Medical University, China (Grant No.20118).

## Conflict of interest

The authors declare that the research was conducted in the absence of any commercial or financial relationships that could be construed as a potential conflict of interest.

## Publisher’s note

All claims expressed in this article are solely those of the authors and do not necessarily represent those of their affiliated organizations, or those of the publisher, the editors and the reviewers. Any product that may be evaluated in this article, or claim that may be made by its manufacturer, is not guaranteed or endorsed by the publisher.
