# Supplementary figures and images for: The relationship between the number of stenotic coronary arteries and the gut microbiome in coronary heart disease patients
Source: Front Cell Infect Microbiol. 2022 Aug 26;12:903828. doi: 10.3389/fcimb.2022.903828 (PMC9458979; doi:10.3389/fcimb.2022.903828)

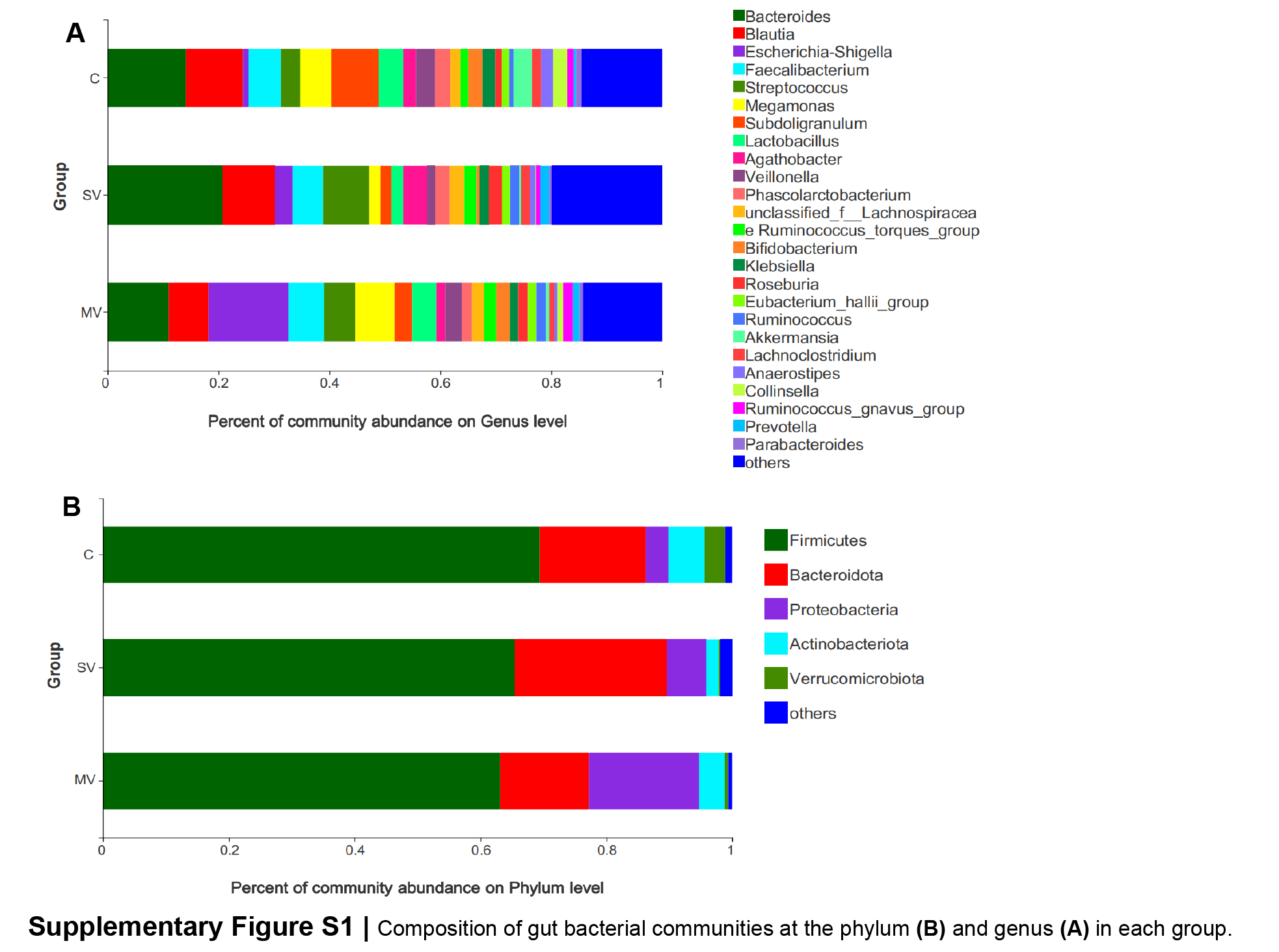

Supplement: Supplementary file 1 [file Image_1.tif]

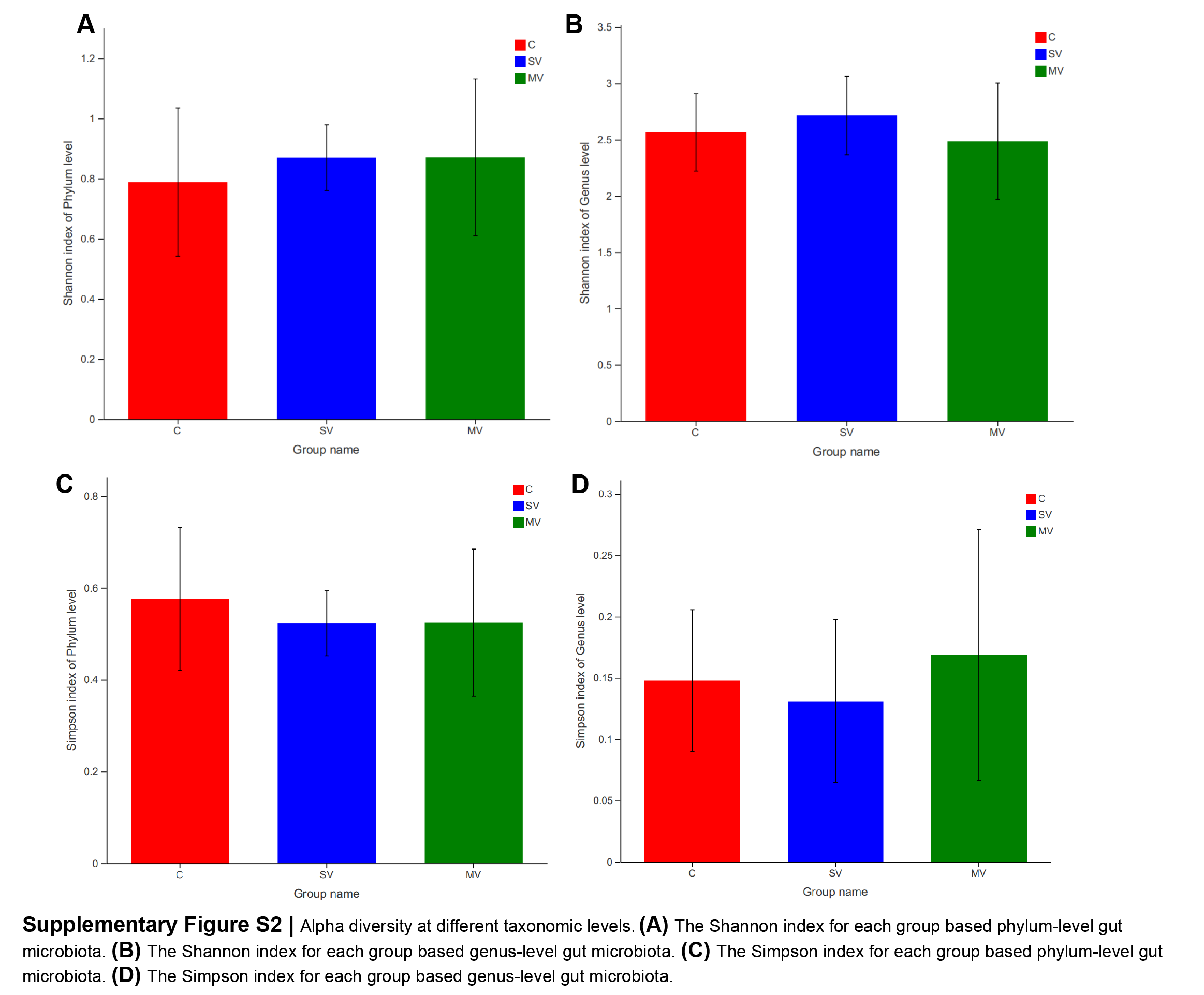

Supplement: Supplementary file 2 [file Image_2.tif]

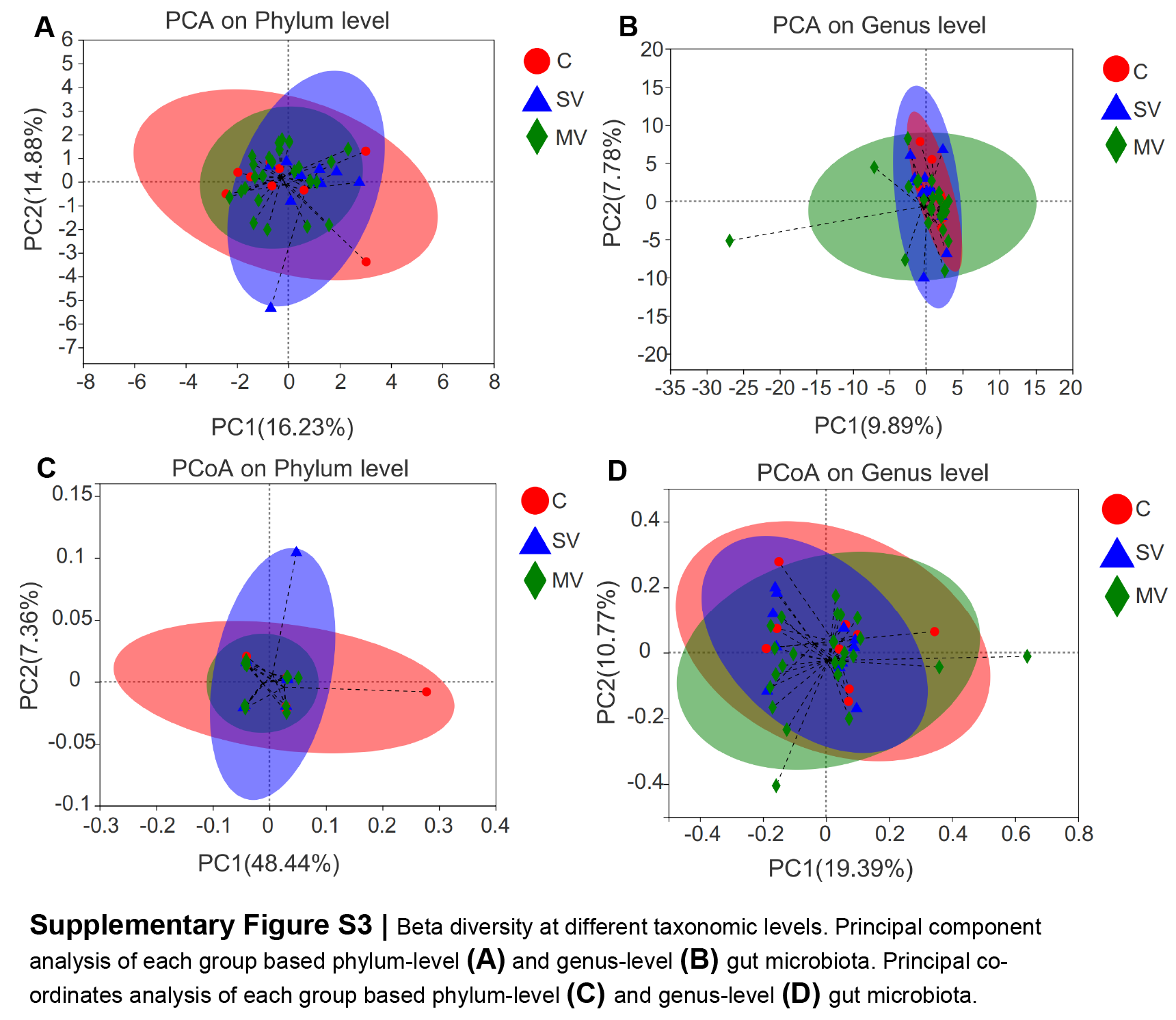

Supplement: Supplementary file 3 [file Image_3.tif]

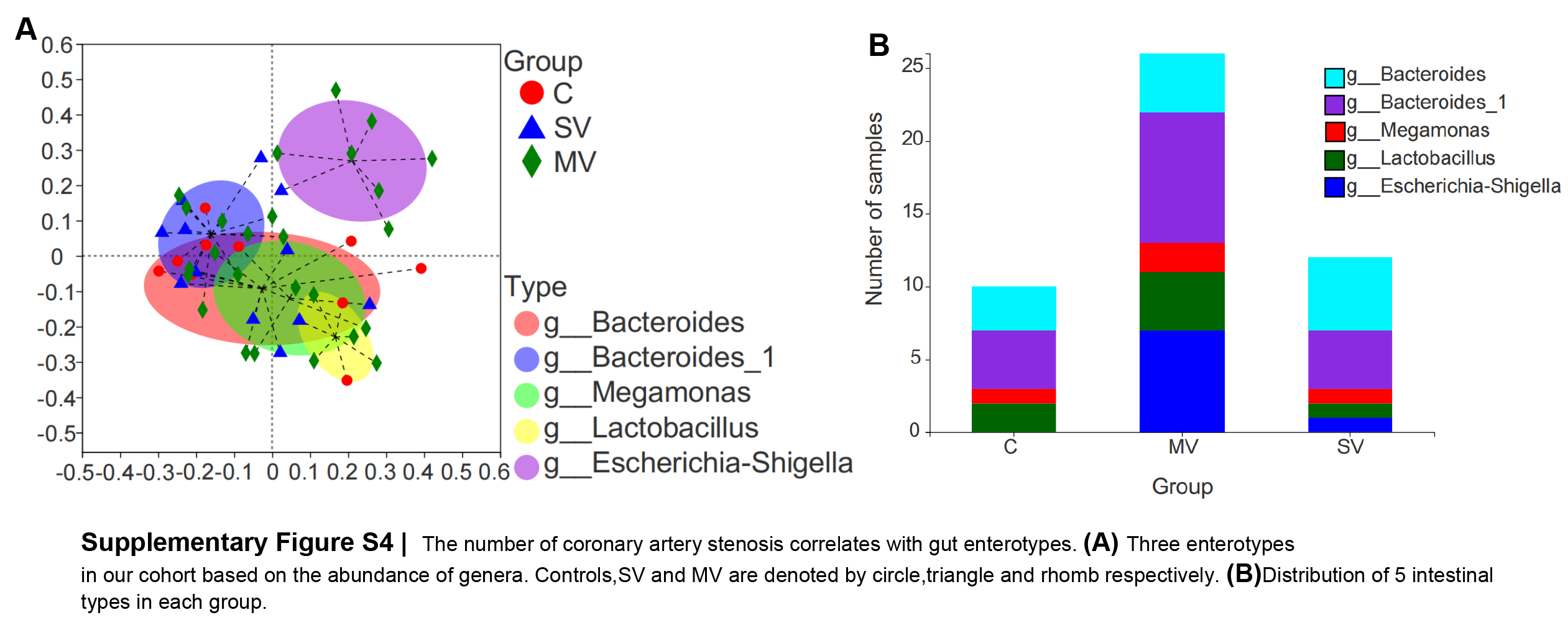

Supplement: Supplementary file 4 [file Image_4.tif]
